# Supplementary material for: Evolutionary game study on multi-agent value co-creation of service-oriented digital transformation in the construction industry
Source: PLoS One. 2023 May 16;18(5):e0285697. doi: 10.1371/journal.pone.0285697 (PMC10187935; doi:10.1371/journal.pone.0285697)
Supplement: S1 File — (ZIP) [file pone.0285697.s001.zip › code.docx]

function dydt=jianzhuye(t,y,I2,I3,I,a,b,L1,L2,C,c,d,u,v,w)

dydt=zeros(3,1);

dydt(1)=y(1)*(1-y(1))*(y(2)*y(3)*(1+w)*a*I+y(3)*(u+v)*c*C-C*c+L1);

dydt(2)=y(2)*(1-y(2))*(y(1)*y(3)*(1+v+w)*b*I+y(1)*v*I2+w*I2-d*C);

dydt(3)=y(3)*(1-y(3))*(y(1)*y(2)*(1-a-b)*I+y(1)*(u+v)*(1-c-d)*C+y(2)*L2+(I3-(1-c-d)*C-L2));

end

clc,clear;

figure（2）;

I2=20,I3=10,I=30,a=1/3,b=1/3,L1=4,L2=6,C=60,c=1/3,d=1/3,u=0.1,v=0.1,w=0.1;

for i=0.1:0.2:1

for j=0.1:0.2:1

for k=0.1:0.2:1

[t,y]=ode45(@(t,y) jianzhuye(t,y,I2,I3,I,a,b,L1,L2,C,c,d,u,v,w),[0 50],[i j k]);

plot3(y(:,1),y(:,2),y(:,3),'linewidth',1);

set(gca,'XTick',[0:0.2:1],'YTick',[0:0.2:1],'ZTick',[0:0.2:1])

hold on

axis([0 1 0 1 0 1])

view([45 10])

end

end

end

grid on

hold on

xlabel('x','Rotation',0);

ylabel('y','Rotation',0);

zlabel('z','Rotation',360,'position',[0 0 1.05]);

clc,clear;

figure（3）;

I2=20,I3=10,I=40,a=1/3,b=1/3,L1=7,L2=6,C=30,c=1/3,d=1/3,u=0.4,v=0.4,w=0.4;

for i=0.1:0.2:1

for j=0.1:0.2:1

for k=0.1:0.2:1

[t,y]=ode45(@(t,y) jianzhuye(t,y,I2,I3,I,a,b,L1,L2,C,c,d,u,v,w),[0 50],[i j k]);

plot3(y(:,1),y(:,2),y(:,3),'linewidth',1);

set(gca,'XTick',[0:0.2:1],'YTick',[0:0.2:1],'ZTick',[0:0.2:1])

hold on

axis([0 1 0 1 0 1])

view([45 10])

end

end

end

grid on

hold on

xlabel('x','Rotation',0);

ylabel('y','Rotation',0);

zlabel('z','Rotation',360,'position',[0 0 1.05]);

clc,clear;

figure（4）;

I2=20,I3=10,I=50,a=1/3,b=1/3,L1=7,L2=7,C=20,c=1/3,d=1/3,u=0.85,v=0.85,w=0.85;

for i=0.1:0.2:1

for j=0.1:0.2:1

for k=0.1:0.2:1

[t,y]=ode45(@(t,y) jianzhuye(t,y,I2,I3,I,a,b,L1,L2,C,c,d,u,v,w),[0 50],[i j k]);

plot3(y(:,1),y(:,2),y(:,3),'linewidth',1);

set(gca,'XTick',[0:0.2:1],'YTick',[0:0.2:1],'ZTick',[0:0.2:1])

hold on

axis([0 1 0 1 0 1])

view([45 10])

end

end

end

grid on

hold on

xlabel('x','Rotation',0);

ylabel('y','Rotation',0);

zlabel('z','Rotation',360,'position',[0 0 1.05]);

clc,clear;

figure(5);

I2=20,I3=10,I=40,a=1/3,b=1/3,L1=7,L2=6,C=30,c=1/3,d=1/3,u=0.4,v=0.4,w=0.4;

[t,y]=ode45(@(t,y) jianzhuye(t,y,I2,I3,I,a,b,L1,L2,C,c,d,u,v,w),[0 1],[0.2 0.2 0.2]);

points=1:1:length(t);

plot(t,y(:,1),'rh-','linewidth',1,'markersize',5,'markerfacecolor','r','markerindices',points);

hold on;

I2=20,I3=10,I=40,a=1/3,b=1/3,L1=7,L2=6,C=30,c=1/3,d=1/3,u=0.4,v=0.4,w=0.4;

[t,y]=ode45(@(t,y) jianzhuye(t,y,I2,I3,I,a,b,L1,L2,C,c,d,u,v,w),[0 1],[0.3 0.3 0.3]);

points=1:1:length(t);

plot(t,y(:,1),'gh-','linewidth',1,'markersize',5,'markerindices',points);

hold on;

I2=20,I3=10,I=40,a=1/3,b=1/3,L1=7,L2=6,C=30,c=1/3,d=1/3,u=0.4,v=0.4,w=0.4;

[t,y]=ode45(@(t,y) jianzhuye(t,y,I2,I3,I,a,b,L1,L2,C,c,d,u,v,w),[0 1],[0.4 0.4 0.4]);

points=1:1:length(t);

plot(t,y(:,1),'bh-','linewidth',1,'markersize',5,'markerindices',points);

hold on;

[t,y]=ode45(@(t,y) jianzhuye(t,y,I2,I3,I,a,b,L1,L2,C,c,d,u,v,w),[0 1],[0.2 0.2 0.2]);

points=1:1:length(t);

plot(t,y(:,2),'ro-','linewidth',1,'markersize',5,'markerfacecolor','r','markerindices',points);

hold on;

I2=20,I3=10,I=40,a=1/3,b=1/3,L1=7,L2=6,C=30,c=1/3,d=1/3,u=0.4,v=0.4,w=0.4;

[t,y]=ode45(@(t,y) jianzhuye(t,y,I2,I3,I,a,b,L1,L2,C,c,d,u,v,w),[0 1],[0.3 0.3 0.3]);

points=1:1:length(t);

plot(t,y(:,2),'go-','linewidth',1,'markersize',5,'markerindices',points);

hold on;

I2=20,I3=10,I=40,a=1/3,1/3,L1=7,L2=6,C=30,c=1/3,d=1/3,u=0.4,v=0.4,w=0.4;

[t,y]=ode45(@(t,y) jianzhuye(t,y,I2,I3,I,a,b,L1,L2,C,c,d,u,v,w),[0 1],[0.4 0.4 0.4]);

points=1:1:length(t);

plot(t,y(:,2),'bo-','linewidth',1,'markersize',5,'markerindices',points);

hold on;

[t,y]=ode45(@(t,y) jianzhuye(t,y,I2,I3,I,a,b,L1,L2,C,c,d,u,v,w),[0 1],[0.2 0.2 0.2]);

points=1:1:length(t);

plot(t,y(:,3),'r--','linewidth',1,'markersize',5,'markerfacecolor','r','markerindices',points);

hold on;

I2=20,I3=10,I=40,a=1/3,b=1/3,L1=7,L2=6,C=30,c=1/3,d=1/3,u=0.4,v=0.4,w=0.4;

[t,y]=ode45(@(t,y) jianzhuye(t,y,I2,I3,I,a,b,L1,L2,C,c,d,u,v,w),[0 1],[0.3 0.3 0.3]);

points=1:1:length(t);

plot(t,y(:,3),'g--','linewidth',1,'markersize',5,'markerindices',points);

hold on;

I2=20,I3=10,I=40,a=1/3,b=1/3,L1=7,L2=6,C=30,c=1/3,d=1/3,u=0.4,v=0.4,w=0.4;

[t,y]=ode45(@(t,y) jianzhuye(t,y,I2,I3,I,a,b,L1,L2,C,c,d,u,v,w),[0 1],[0.4 0.4 0.4]);

points=1:1:length(t);

plot(t,y(:,3),'b--','linewidth',1,'markersize',5,'markerindices',points);

hold on;

set(0,'defaultfigurecolor','w')

grid on

hold on

xlabel('$Time$','interpreter','latex','Rotation',0);

ylabel('$Proportion$','interpreter','latex');

set(gca,'XTick',[0:0.2:1],'YTick',[0:0.2:1])

axis([0 1 0 1]);

legend('x=0.2','x=0.3','x=0.4','y=0.2','y=0.3','y=0.4','z=0.2','z=0.3','z=0.4');

clc,clear;

figure(6);

%u=0.3

u=0.3,I2=20,I3=10,I=40,a=1/3,b=1/3,L1=7,L2=6,C=30,c=1/3,d=1/3,v=0.4,w=0.4;

[t,y]=ode45(@(t,y) jianzhuye(t,y,I2,I3,I,a,b,L1,L2,C,c,d,u,v,w),[0 50],[0.2 0.2 0.2]);

plot3(y(:,1),y(:,2),y(:,3),'r+','linewidth',1);

hold on ;

%u=0.4

u=0.4,I2=20,I3=10,I=40,a=1/3,b=1/3,L1=7,L2=6,C=30,c=1/3,d=1/3,v=0.4,w=0.4;

[t,y]=ode45(@(t,y) jianzhuye(t,y,I2,I3,I,a,b,L1,L2,C,c,d,u,v,w),[0 50],[0.2 0.2 0.2]);

plot3(y(:,1),y(:,2),y(:,3),'g-','linewidth',1);

hold on ;

%u=0.7

u=0.7,I2=20,I3=10,I=40,a=1/3,b=1/3,L1=7,L2=6,C=30,c=1/3,d=1/3,v=0.4,w=0.4;

[t,y]=ode45(@(t,y) jianzhuye(t,y,I2,I3,I,a,b,L1,L2,C,c,d,u,v,w),[0 50],[0.2 0.2 0.2]);

plot3(y(:,1),y(:,2),y(:,3),'b--','linewidth',1);

hold on ;

set(gca,'XTick',[0:0.2:1],'YTick',[0:0.2:1],'ZTick',[0:0.2:1])

axis([0 1 0 1 0 1])

xlabel('$x$','interpreter','latex');ylabel('$y$','interpreter','latex');zlabel('$z$','interpreter','latex','Rota

tion',360,'position',[-0.1 1 1.1]);

grid on

hold on

set(0,'defaultfigurecolor','w')

legend({'{\it\fontname{Bodoni MT}u}=0.3','{\it\fontname{Bodoni

MT}u}=0.4','{\it\fontname{Bodoni MT}u}=0.7'},'location','northeast');

text(0.4 ,0.2 ,0.3,'$ESS$','interpreter','latex');

annotation('arrow',[0.55 0.35],[0.35 0.32]);

annotation('arrow',[0.58 0.58],[0.38 0.45]);

axes('position',[0.13 0.32 0.2 0.2]);

u=0.3,I2=20,I3=10,I=40,a=1/3,b=1/3,L1=7,L2=6,C=30,c=1/3,d=1/3,v=0.4,w=0.4;

[t,y]=ode45(@(t,y) jianzhuye(t,y,I2,I3,I,a,b,L1,L2,C,c,d,u,v,w),[0 50],[0.2 0.2 0.2]);

plot3(y(:,1),y(:,2),y(:,3),'r+','linewidth',1);

hold on

u=0.4,I2=20,I3=10,I=40,a=1/3,b=1/3,L1=7,L2=6,C=30,c=1/3,d=1/3,v=0.4,w=0.4;

[t,y]=ode45(@(t,y) jianzhuye(t,y,I2,I3,I,a,b,L1,L2,C,c,d,u,v,w),[0 50],[0.2 0.2 0.2]);

plot3(y(:,1),y(:,2),y(:,3),'g-','linewidth',1);

hold on

u=0.7,I2=20,I3=10,I=40,a=1/3,b=1/3,L1=7,L2=6,C=30,c=1/3,d=1/3,v=0.4,w=0.4;

[t,y]=ode45(@(t,y) jianzhuye(t,y,I2,I3,I,a,b,L1,L2,C,c,d,u,v,w),[0 50],[0.2 0.2 0.2]);

plot3(y(:,1),y(:,2),y(:,3),'b--','linewidth',1);

hold on

set(gca,'XTick',[0:0.1:0.5],'YTick',[0:0.1:0.5],'ZTick',[0:0.1:0.5])

axis([0 0.5 0 0.5 0 0.5])

xlabel('$x$','interpreter','latex');ylabel('$y$','interpreter','latex','Rotation',360,'position',[-0.1 1 1.1]);

grid on

hold on

set(0,'defaultfigurecolor','w')

view([0 90]);

xlabel('x','position',[0.8 1 0.3])

ylabel('y','position',[0.1 1 0.8],'Rotation',360)

clc,clear;

figure(7);

%v=0.3

v=0.3,I2=20,I3=10,I=40,a=1/3,b=1/3,L1=7,L2=6,C=30,c=1/3,d=1/3,u=0.4,w=0.4;

[t,y]=ode45(@(t,y) jianzhuye(t,y,I2,I3,I,a,b,L1,L2,C,c,d,u,v,w),[0 50],[0.2 0.2 0.2]);

plot3(y(:,1),y(:,2),y(:,3),'r+','linewidth',1);

hold on ;

%v=0.4

v=0.4,I2=20,I3=10,I=40,a=1/3,b=1/3,L1=7,L2=6,C=30,c=1/3,d=1/3,u=0.4,w=0.4;

[t,y]=ode45(@(t,y) jianzhuye(t,y,I2,I3,I,a,b,L1,L2,C,c,d,u,v,w),[0 50],[0.2 0.2 0.2]);

plot3(y(:,1),y(:,2),y(:,3),'g-','linewidth',1);

hold on ;

%v=0.7

v=0.7,I2=20,I3=10,I=40,a=1/3,b=1/3,L1=7,L2=6,C=30,c=1/3,d=1/3,u=0.4,w=0.4;

[t,y]=ode45(@(t,y) jianzhuye(t,y,I2,I3,I,a,b,L1,L2,C,c,d,u,v,w),[0 50],[0.2 0.2 0.2]);

plot3(y(:,1),y(:,2),y(:,3),'b--','linewidth',1);

hold on ;

set(gca,'XTick',[0:0.2:1],'YTick',[0:0.2:1],'ZTick',[0:0.2:1])

axis([0 1 0 1 0 1])

xlabel('$x$','interpreter','latex');ylabel('$y$','interpreter','latex');zlabel('$z$','interpreter','latex','Rota

tion',360,'position',[-0.1 1 1.1]);

grid on

hold on

set(0,'defaultfigurecolor','w')

legend({'{\it\fontname{Bodoni MT}v}=0.3','{\it\fontname{Bodoni

MT}v}=0.4','{\it\fontname{Bodoni MT}v}=0.7'},'location','northeast');

text(0.4 ,0.2 ,0.3,'$ESS$','interpreter','latex');

annotation('arrow',[0.55 0.35],[0.35 0.32]);

annotation('arrow',[0.58 0.58],[0.38 0.45]);

axes('position',[0.13 0.32 0.2 0.2]);

v=0.3,I2=20,I3=10,I=40,a=1/3,b=1/3,L1=7,L2=6,C=30,c=1/3,d=1/3,u=0.4,w=0.4;

[t,y]=ode45(@(t,y) jianzhuye(t,y,I2,I3,I,a,b,L1,L2,C,c,d,u,v,w),[0 50],[0.2 0.2 0.2]);

plot3(y(:,1),y(:,2),y(:,3),'r+','linewidth',1);

hold on

v=0.4,I2=20,I3=10,I=40,a=1/3,b=1/3,L1=7,L2=6,C=30,c=1/3,d=1/3,u=0.4,w=0.4;

[t,y]=ode45(@(t,y) jianzhuye(t,y,I2,I3,I,a,b,L1,L2,C,c,d,u,v,w),[0 50],[0.2 0.2 0.2]);

plot3(y(:,1),y(:,2),y(:,3),'g-','linewidth',1);

hold on

v=0.7,I2=20,I3=10,I=40,a=1/3,b=1/3,L1=7,L2=6,C=30,c=1/3,d=1/3,u=0.4,w=0.4;

[t,y]=ode45(@(t,y) jianzhuye(t,y,I2,I3,I,a,b,L1,L2,C,c,d,u,v,w),[0 50],[0.2 0.2 0.2]);

plot3(y(:,1),y(:,2),y(:,3),'b--','linewidth',1);

hold on

set(gca,'XTick',[0:0.1:0.5],'YTick',[0:0.1:0.5],'ZTick',[0:0.1:0.5])

axis([0 0.5 0 0.5 0 0.5])

xlabel('$x$','interpreter','latex');ylabel('$y$','interpreter','latex','Rotation',360,'position',[-0.1 1 1.1]);

grid on

hold on

set(0,'defaultfigurecolor','w')

view([0 90]);

xlabel('x','position',[0.8 1 0.3])

ylabel('y','position',[0.1 1 0.8],'Rotation',360)

clc,clear;

figure(8);

%w=0.3

w=0.3,I2=20,I3=10,I=40,a=1/3,b=1/3,L1=7,L2=6,C=30,c=1/3,d=1/3,u=0.4,v=0.4;

[t,y]=ode45(@(t,y) jianzhuye(t,y,I2,I3,I,a,b,L1,L2,C,c,d,u,v,w),[0 50],[0.2 0.2 0.2]);

plot3(y(:,1),y(:,2),y(:,3),'r+','linewidth',1);

hold on ;

%w=0.4

w=0.4,I2=20,I3=10,I=40,a=1/3,b=1/3,L1=7,L2=6,C=30,c=1/3,d=1/3,u=0.4,v=0.4;

[t,y]=ode45(@(t,y) jianzhuye(t,y,I2,I3,I,a,b,L1,L2,C,c,d,u,v,w),[0 50],[0.2 0.2 0.2]);

plot3(y(:,1),y(:,2),y(:,3),'g-','linewidth',1);

hold on ;

%w=0.7

w=0.7,I2=20,I3=10,I=40,a=1/3,b=1/3,L1=7,L2=6,C=30,c=1/3,d=1/3,u=0.4,v=0.4;

[t,y]=ode45(@(t,y) jianzhuye(t,y,I2,I3,I,a,b,L1,L2,C,c,d,u,v,w),[0 50],[0.2 0.2 0.2]);

plot3(y(:,1),y(:,2),y(:,3),'b--','linewidth',1);

hold on ;

set(gca,'XTick',[0:0.2:1],'YTick',[0:0.2:1],'ZTick',[0:0.2:1])

axis([0 1 0 1 0 1])

xlabel('$x$','interpreter','latex');ylabel('$y$','interpreter','latex');zlabel('$z$','interpreter','latex','Rota

tion',360,'position',[-0.1 1 1.1]);

grid on

hold on

set(0,'defaultfigurecolor','w')

legend({'{\it\fontname{Bodoni MT}w}=0.3','{\it\fontname{Bodoni

MT}w}=0.4','{\it\fontname{Bodoni MT}w}=0.7'},'location','northeast');

text(0.4 ,0.2 ,0.3,'$ESS$','interpreter','latex');

annotation('arrow',[0.55 0.35],[0.35 0.32]);

annotation('arrow',[0.58 0.58],[0.38 0.45]);

axes('position',[0.13 0.32 0.2 0.2]);

w=0.3,I2=20,I3=10,I=40,a=1/3,b=1/3,L1=7,L2=6,C=30,c=1/3,d=1/3,u=0.4,v=0.4;

[t,y]=ode45(@(t,y) jianzhuye(t,y,I2,I3,I,a,b,L1,L2,C,c,d,u,v,w),[0 50],[0.2 0.2 0.2]);

plot3(y(:,1),y(:,2),y(:,3),'r+','linewidth',1);

hold on

w=0.4,I2=20,I3=10,I=40,a=1/3,b=1/3,L1=7,L2=6,C=30,c=1/3,d=1/3,u=0.4,v=0.4;

[t,y]=ode45(@(t,y) jianzhuye(t,y,I2,I3,I,a,b,L1,L2,C,c,d,u,v,w),[0 50],[0.2 0.2 0.2]);

plot3(y(:,1),y(:,2),y(:,3),'g-','linewidth',1);

hold on

w=0.7,I2=20,I3=10,I=40,a=1/3,b=1/3,L1=7,L2=6,C=30,c=1/3,d=1/3,u=0.4,v=0.4;

[t,y]=ode45(@(t,y) jianzhuye(t,y,I2,I3,I,a,b,L1,L2,C,c,d,u,v,w),[0 50],[0.2 0.2 0.2]);

plot3(y(:,1),y(:,2),y(:,3),'b--','linewidth',1);

hold on

set(gca,'XTick',[0:0.2:1],'YTick',[0:0.2:1],'ZTick',[0:0.2:1])

axis([0 1 0 1 0 1])

xlabel('$x$','interpreter','latex');ylabel('$y$','interpreter','latex','Rotation',360,'position',[-0.1 1 1.1]);

grid on

hold on

set(0,'defaultfigurecolor','w')

view([0 90]);

xlabel('x','position',[0.8 1 0.3])

ylabel('y','position',[0.1 1 0.8],'Rotation',360)
